# Is postnatal acetaminophen therapy problematic for preterm infants?

**DOI:** 10.1038/s41390-024-03204-5

**Published:** 2024-04-10

**Authors:** Richard J. Martin, Anna Maria Hibbs

**Affiliations:** Division of Neonatology Rainbow Babies and Children’s Hospital, 11100 Euclid Ave, Cleveland, OH 44106 USA

In this issue, Srajer et al. report on the neurodevelopmental outcomes at 18–21 months of preterm infants treated with acetaminophen in the NICU.^1^ Their study shows no evidence of neurological harm from acetaminophen, after adjusting for known risk factors for neurodevelopmental impairment (NDI). Temporal trends in both pain and patent ductus arteriosus (PDA) management have led to an increase in acetaminophen use in the NICU in recent years, resulting in a crucial need for long-term safety data in this vulnerable population.

Despite our best efforts to minimize interventions, preterm infants are necessarily exposed to multiple invasive and painful procedures. Given appropriate reluctance to administer narcotics such as morphine for pain relief, acetaminophen (also known as paracetamol) seems like an acceptable alternative. The American Academy of Pediatrics policy statement on procedural pain in neonates described acetaminophen as a promising therapy to decrease post-operative morphine.^2^ It is certainly widely used in both pediatric and adult populations to treat both moderate pain and fever, but robust long-term safety data for acetaminophen for pain relief for preterm NICU patients is needed.

The use of acetaminophen to close PDAs is relatively recent. Hammerman made the surprising observation that paracetamol given to a preterm infant for unrelated reasons happened to result in PDA closure^.^^3^ The concept that PDA closure might improve respiratory function was introduced in the mid to late 1970’s.^4,5^ This was followed by multiple studies exploring the promise of pharmacologic closure via cyclooxygenase inhibition. The largest of these trials performed two decades after the original observations showed that prophylactic indomethacin reduced the frequency of a PDA and severe intraventricular hemorrhage but did not improve survival without neurosensory impairment.^6^ Given that PDA closure would reduce excessive left to right shunting and pulmonary blood flow it seemed possible that more prolonged respiratory morbidity might be reduced. Unfortunately a large retrospective cohort study from the NICHD Neonatal Research Network showed no benefit of prophylactic indomethacin on the incidence of bronchopulmonary dysplasia (BPD)^.^^7^ In a very recent multicenter noninferiority trial extremely preterm infants with an echocardiographically confirmed PDA were randomized to early ibuprofen or expectant management.^8^ Expectant management was noninferior as their primary outcome, however secondary analysis revealed a higher incidence of moderate to severe BPD in the early ibuprofen treated group (51 vs 33%), echoing concerns about increased BPD in infants without PDA who received indomethacin prophylaxis in the TIPP trial.^9^ This raises speculation regarding potential respiratory morbidity associated with pharmacotherapy, especially given variability in dosing and duration of therapy as well as given the lack of evidence of benefit for PDA closure.

Subsequent impetus for an alternative medical intervention for PDA closure in the form of acetaminophen was initially driven by concern for the side effects of indomethacin, primarily renal and gastrointestinal. However, safety of paracetamol in this role and dosage was recognized as uncertain and mechanism of action beyond cyclooxygenase inhibition unclear.^10^ The potential for hepatotoxicity has been a potential concern as acetaminophen metabolism by cytochrome p450 could induce toxic metabolites. It has been speculated that pulmonary expression of the cytochrome system could contribute to longer term respiratory morbidity in the face of acetaminophen exposure during a window of increased developmental susceptibility.^11^ Interestingly, an analysis of acetaminophen metabolites after increasing exposure did note a gestational age dependent change in a glucuronidation pathway the relevance of which is not clear^12^ In addition, a growing number of non-definitive studies suggesting a link between in utero acetaminophen exposure and neurodevelopmental impairment have heightened the need for safety data in preterm infants at similar developmental stages. Thus studies exploring safety of acetaminophen administration to preterm infants should be a high priority, especially give its widespread use in the NICU.

Thus, the report by Srajer et al. is an important step in ensuring the safety and efficacy of acetaminophen in preterm infants. These reassuring data are consistent with a recent report in small trial of very preterm infants that paracetamol did not induce adverse morbidities at five years of age.^13^ Therefore randomized trials of acetaminophen for pain control assessing long-term outcomes are needed, as are analyses by drug class in trials of PDA medical management. In addition, with compelling questions about the respiratory effects of acetaminophen, future studies are needed to assess respiratory outcomes as well as NDI.
